# Solid Particle Swarm Measurement in Jet Fuel Based on Mie Scattering Theory and Extinction Method

**DOI:** 10.3390/s23052837

**Published:** 2023-03-05

**Authors:** Limin He, Heng Wu, Jifeng Li, Bingqiang Li, Yulai Sun, Peng Jiang, Xiaoxu Wang, Guanyu Lin

**Affiliations:** 1 Changchun Institute of Optics, Fine Mechanics and Physics, Chinese Academy of Sciences, Changchun 130033, China; 2 University of Chinese Academy of Sciences, Beijing 100049, China

**Keywords:** jet fuel, particle size measurement, dynamic scattering, mass concentration measurement

## Abstract

To overcome the disadvantages of small and random samples in static detection, this paper presents a study on dynamic measurements of solid particles in jet fuel using large samples. In this paper, the Mie scattering theory and Lambert-Beer law are used to analyze the scattering characteristics of copper particles in jet fuel. We have presented a prototype for multi-angle scattered and transmitted light intensity measurements of particle swarms in jet fuel which is used to test the scattering characteristics of the jet fuel mixture with 0.5–10 μm particle sizes and 0–1 mg/L concentrations of copper particles. The vortex flow rate was converted to an equivalent pipe flow rate using the equivalent flow method. Tests were conducted at equivalent flow rates of 187, 250 and 310 L/min. Through numerical calculations and experiments, it has been discovered that the intensity of the scattering signal decreases as the scattering angle increases. Meanwhile, both the scattered light intensity and transmitted light intensity would vary with the particle size and mass concentration. Finally, the relationship equation between light intensity and particle parameters has also been summarized in the prototype based on the experimental results, which proves its detection capability.

## 1. Introduction

In aerospace applications, jet fuel needs to be continuously burned at a low temperature and low pressure. It is not only used in turbo and piston engines but also in rocket engines, which are the heart of the aircraft. The main failure mode of engines is abrasion, and impurity particles are the main cause of it. It is generally accepted that there should not be too many impurity particles in jet fuel, as this will lead to mechanical failure. Therefore, the cleanliness of jet fuel is essential for ensuring aviation safety [1,2].

Jet fuel can be produced by refining crude oil, which entails a series of fractional distillations under atmosphere and vacuum [3,4]. This refining process often takes place in plants far from cities, while jet fuel is often used around cities, resulting in a long period of multiple transportation processes from production to use [5]. During long distance transportation, jet fuel would pass through pipelines, trains, tanker trucks and oil tankers before it can be injected into aircraft tanks by refueling tender or apron refueling systems [6]. At the same time, shedding of metal shavings, metal oxides, and sealing materials from storage devices and pipes can contaminate jet fuel [7]. In summary, jet fuel can be contaminated with impurity particles during storage, transportation and refilling [8].

Common types of solid particles in jet fuel are dust, metal shavings, silica, fibrous materials, elastomeric materials, hydrocarbon/oxidation materials including rust, and any other solid matter [9]. The sediment in jet fuel is divided into coarse sediment and fine sediment. The particle size of coarse sediment is more than 10 µm, while that of fine sediment is less than 10 µm. For jet fuel, the maximum limit of particle mass concentration is 1 mg/L for avoiding engine failure [9].

The continuous impact of solid particles in the sophisticated engine will increase the abrasion of engine assemblies and parts, resulting in uneven pressure across the turbine [10]. In severe cases, there may be cracks and even breakages at the root of the turbine blades. Solid particles can also damage the sealing of kinematic pairs such as the plunger piston and plunger sleeve of the injection pump, the needle valve, and the valve body of the injector. Precipitation of solid particles causes clogging and sticking of precision components such as fuel injectors and high-pressure pumps [6,10]. In the high temperature zone, impurity particles can catalyze the oxidation reaction of the fuel, increase the corrosion of the fuel equipment, and cause more electrostatic buildup during refilling [11]. Solid impurity particles also have a greater impact on the fuel supply system, which increases the consumption of jet fuel, deteriorates the engine starting performance, and leads to abnormal fuel supply in a serious condition [12,13,14].

Automatic particle counters based on extinction, scattering or imaging are often used to detect particles in fuel [15,16,17,18,19,20,21]. Yuki Kuruma has combined particle counting and mass measurement in a static detection method to determine the concentration of particles in liquids [22]. The particle counters show great potential for obtaining the shape and size distribution of impurity particles, but they can only detect small samples that under 3 L/min. The sample is also affected by randomness, which is unable to detect large samples [23]. The image detection method can obtain the shape characteristics of the particles, but the processing data volume is too large and the sample must be smaller that about 10 mL/min [23,24,25]. In contrast, using scatter theory for the detection of 0.5–10 µm particles has the potential to enable dynamic measurements of large samples. The method of using a rotating measurement system instead of a circulating flow measurement system is proposed. The measurement errors caused by particles sedimentation can be avoided, and simulate different flow rates between 187 and 310 L/min. It effectively reduces the complexity of the system, improves the measurement efficiency, and facilitates the measurement of multiple particle impurities.

In this paper, based on Mie scattering theory and Lambert-Beer law, the scattering characteristics of a copper particle swarm in jet fuel have been studied by numerical simulations. A prototype for detecting impurity particles in jet fuel has created. Using the prototype, the dynamic measurement method for large samples of impurity particles size and mass concentration in jet fuel has been studied using copper particles as samples. The experiments have obtained results similar to the theoretical analysis, and finally, the rules for the variation of scattered light intensity and transmitted light intensity with particle properties obtained from the experiments have been summarized and the corresponding empirical formulas have been given.

## 2. Materials and Methods

### 2.1. Experimental Method

When particles were exposed to light, they would scatter and absorb it. Light scattering was handled by Mie scattering theory, and light absorption was handled by Lambert-Beer law. Particle swarm with different particle sizes and mass concentration scatters and absorbs different light energy [26,27]. We used this principle to study the effect of different particle sizes and mass concentrations on scattered and transmitted light.

**Figure 1:**
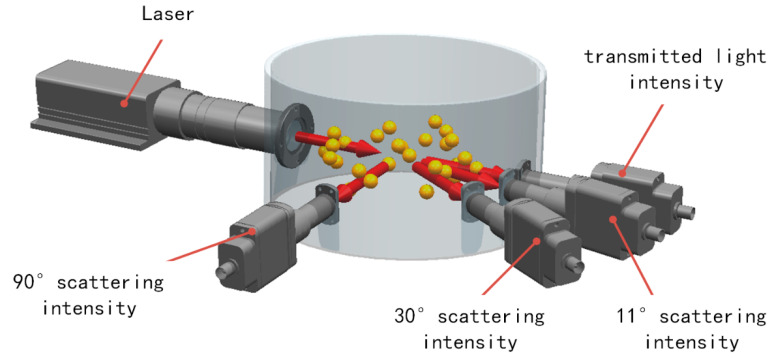
Multi-angle scattering principle prototype schematic diagram (out of proportion).

Then the basic structure of the detection system was constructed. Figure 1 was the multi-angle scattering prototype schematic diagram. It should be noted here that it is out of proportion. A parallel near infrared laser with a wavelength of 940 nm was used as the light source, and the sample pool was cylindrical in shape with a diameter of 150 mm to match the shape of the jet fuel refueling pipe. The intersection of the parallel beam with a diameter of 10 mm sent by the laser and the sample pool was the optical path. The length of the optical path was about 150 mm. The flat window glass was used in order to keep the direction of light propagation constant after passing through it. The energy of the scattered light received at different angles should be different, so three scattering angles of 11°, 30° and 90° and one transmission angle were selected. The reasons for choosing the above three angles were as follows: as the scattered light intensity gradually decreases with the scattering angle, 11° was the angle at which the scattered light intensity was the strongest but not affected by the light passing through; 30° was the angle at which the light intensity decays; and 90° was the angle at which the light intensity tends to stabilize. In Section 3, we simulated and analyzed the law of scattered light energy and transmitted light energy of copper particles with different particle sizes and mass concentrations at multiple angles. After the simulation analysis, a prototype of the multi-angle measurement system was designed. The specific design of the prototype was in Section 4.

### 2.2. Theory Method

The Mie scattering theory was applicable to the case that the spherical particle size is approximately equal to the wavelength. The wavelength of the laser used in this paper was 940 nm and the detected particle size was 0.5–10 µm, therefore, the intensity of light scattered by impurity particles in the jet fuel in this paper can be calculated using the Mie scattering theory. When a spherical particle is illuminated by collimated light, the particle can scatter part of the light energy. Light energy can be detected at different scattering angles. The relationships between the scattered light intensity $I_{s}\left(\theta \right)$ of the particles on the observation points and the initial incident light intensity $I_{0}$ can be expressed using Mie scattering theory [28,29]. When the incident light is fully polarized, with the particle as the origin, the incident light is directed along the *z*-axis and the electric vector is directed along the *x*-axis, the scattered light intensity $I_{s}\left(\theta \right)$ at the point P(r,θ,φ)is:

(1) $$I_{s}\left(\theta \right)=\frac{\lambda^{2}}{4\pi^{2}r^{2}}I_{0}\left[i_{1}\left(d,m,\theta \right)\sin^{2}\varphi +i_{2}\left(d,m,\theta \right)\cos^{2}\varphi \right]$$

where $I_{0}$ is the initial incident light intensity, λ is the wavelength of the laser, *r* is the distance between the particle and the observation point, $i_{1}\left(d,m,\theta \right)$ and $i_{2}\left(d,m,\theta \right)$ are the intensity functions of the scattered light in the vertical and horizontal directions, *d* is the particle size, *m* is the relative refractive index—the ratio of the particle refractive index *m*_2_ to the medium refractive index *m*_1_. θ is the scattering angle of the incident light after scattering by particles, and φ is the angle between the vibration surface of the incident light and the scattering surface.

Introducing the amplitude equation $s_{q}\left(d,m,\theta \right)$(where *q* = 1 or 2), $i_{q}\left(d,m,\theta \right)={\left|s_{q}\left(d,m,\theta \right)\right|}^{2}$.

The amplitude functions $s_{1}\left(d,m,\theta \right)$ and $s_{2}\left(d,m,\theta \right)$ are an infinite series consisting of the Bessel function and the Legendre function, and they can be represented as:

(2) $$s_{1}\left(d,m,\theta \right)=\sum_{n=1}^{\infty }\frac{2n+1}{n\left(n+1\right)}\left(a_{n}\pi_{n}+b_{n}\tau_{n}\right)$$

(3) $$s_{2}\left(d,m,\theta \right)=\sum_{n=1}^{\infty }\frac{2n+1}{n\left(n+1\right)}\left(a_{n}\tau_{n}+b_{n}\pi_{n}\right)$$

where $a_{n}$ and $b_{n}$ are called the Mie scattering coefficient, which are the core of Mie scattering calculation, and are related to the refractive index *m* and dimensionless particle size parameter α. Where $\pi_{n}$ and $\tau_{n}$ are related to the scattering angle θ.

The expressions for $\pi_{n}$ and $\tau_{n}$ are:

(4) $$\pi_{n}=\frac{P_{n}^{\left(1\right)}\left(\cos\;\theta \right)}{\sin\;\theta }=\frac{dP_{n}\left(\cos\;\theta \right)}{\mathrm{dcos}\;\theta }$$

(5) $$\tau_{n}=\frac{dP_{n}^{\left(1\right)}\left(\cos\;\theta \right)}{d\theta }$$

$P_{n}\left(\cos\;\theta \right)$ is the Legendre function of cos θ,

$P_{n}^{\left(1\right)}\left(\cos\;\theta \right)$ is the first kind of association Legendre function of first-order to the nth power with respect to cos θ, The expressions for $a_{n}$ and $b_{n}$ are:

(6) $$a_{n}=\frac{\psi_{n}\left(\alpha \right)\psi_{n}^{\prime }\left(m\alpha \right)-m\psi_{n}^{\prime }\left(\alpha \right)\psi_{n}\left(m\alpha \right)}{\xi_{n}\left(\alpha \right)\psi_{n}^{\prime }\left(m\alpha \right)-m\xi_{n}^{\prime }\left(\alpha \right)\psi_{n}\left(m\alpha \right)}$$

(7) $$b_{n}=\frac{m\psi_{n}\left(\alpha \right)\psi_{n}^{\prime }\left(m\alpha \right)-\psi_{n}^{\prime }\left(\alpha \right)\psi_{n}\left(m\alpha \right)}{m\xi_{n}\left(\alpha \right)\psi_{n}^{\prime }\left(m\alpha \right)-\xi_{n}^{\prime }\left(\alpha \right)\psi_{n}\left(m\alpha \right)}$$

where *α* is a dimensionless parameter of particle size. The formula is$\;\alpha =m_{1}\pi d/\lambda$, where $m_{1}$ is the complex refractive index of the medium, *d* is the particle size and λ is the wavelength. $\psi_{n}\left(z\right)$ and $\xi_{n}\left(z\right)$ are Ricatti-Bessel functions, which are functions of the semi-integer order Bessel function and the Hankel function of the second kind. They can be written as:

(8) $$\psi_{n}\left(z\right)={\left(\frac{\pi z}{2}\right)}^{\frac{1}{2}}J_{n+\frac{1}{2}}\left(z\right)$$

(9) $$\xi_{n}\left(z\right)={\left(\frac{\pi z}{2}\right)}^{\frac{1}{2}}H_{n+\frac{1}{2}}^{\left(2\right)}\left(z\right)={\left(\frac{\pi z}{2}\right)}^{\frac{1}{2}}\left[J_{n+\frac{1}{2}}\left(z\right)-i*Y_{n+\frac{1}{2}}\left(z\right)\right]$$

$J_{n+\frac{1}{2}}\left(z\right)$ and $Y_{n+\frac{1}{2}}\left(z\right)$ are Bessel functions of the first and second kinds of semi-integer order.

**Figure 2:**
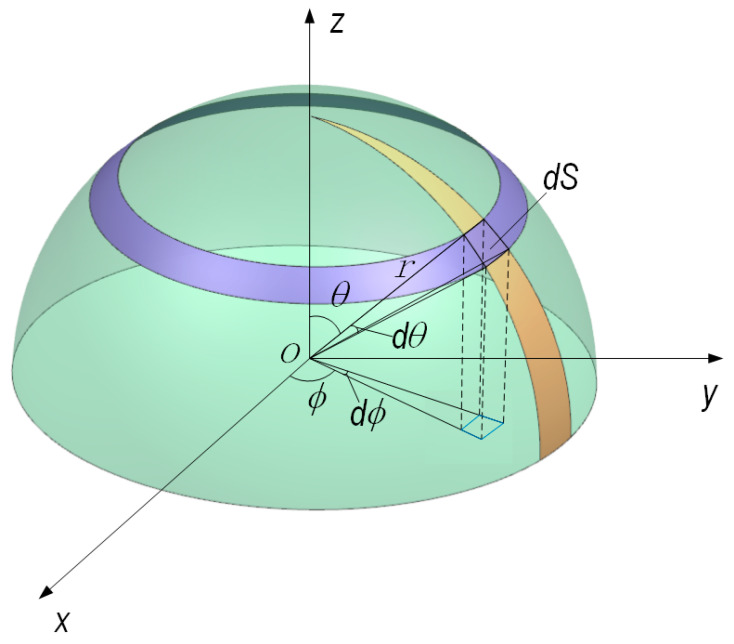
Schematic diagram of light flux calculation.

As shown in Figure 2, when the receiving area is flat, the relationships between the light flux *F* and the initial incident light intensity $I_{0}$ can be calculated using the light flux calculation formula of Mie scattering.

The light flux F is calculated using a double integral, and the F can be described as:

(10) $$F=\frac{\lambda^{2}I_{0}}{4\pi^{2}}\int_{\varphi_{0}-\Delta \varphi }^{\varphi_{0}+\Delta \varphi }\int_{\theta_{1}}^{\theta_{2}}\left(i_{1}\sin^{2}\varphi +i_{2}\cos^{2}\varphi \right)\sin\;\theta d\theta d\varphi$$

Light flux for discrete particle swarm can be written as:

(11) $$F=\frac{\lambda^{2}I_{0}}{4\pi^{2}}\sum_{j=1}^{n}\int_{\theta_{1}}^{\theta_{2}}\left[\left(i_{1}+i_{2}\right)\Delta \varphi -\frac{i_{1}-i_{2}}{2}\cos2\varphi_{0}\sin2\Delta \varphi \right]\sin\;\theta d\theta$$

The Lambert-Beer law can describe the extinction of light by particles. The theory is applied to homogeneous solutions and its upper limit for particle size is 10 µm and the lower limit is 10 nm. The Lambert-Beer law is used to describe the relationships between the transmitted light intensity *I* and the initial light intensity $I_{0}\;$[30]:

(12) $$I=I_{0}e^{-Nk_{ext}l\frac{\pi d^{2}}{4}}$$

where *N* represents the number of particles with the same particle size and are distributed irregularly in the unit volume, $k_{ext}$ is the extinction coefficient of the particles, *l* is the thickness of the homogeneous solution, and *d* is the size of the particles.

### 2.3. Experimental Material

The experimental materials required for this experiment were mainly copper particle (Bu Wei Applied Material Technology Co. Ltd., Shanghai, China) jet fuel(RP3) mixtures of different mass concentrations. Because copper material is soft and easy to fall off, and standard specifications of copper particles are easy to obtain. In the mixing of the two components, the two were first formulated into a homogeneous and highly concentrated mixture using an ultrasonic vibrator. Subsequently, a certain amount of the concentrated mixture was added to the sample pool using a pipette gun to obtain a mixture of jet fuel and impurities at the target concentration. Copper particles with average particle size of 500 nm, 1 µm, 3 µm, 5 µm and 10 µm were selected as test samples.

## 3. Simulation Processes and Results

### 3.1. Calculation of Complex Refractive Index for Jet Fuel

Jet fuel is a kind of dissipative medium that absorbs light. The refractive index of the dissipative medium can be expressed as m=n−iη, and the real part *n* is the scattering coefficient of the medium which shows the refraction property. η is the absorption coefficient of the medium, which represents the absorption property. The values of the real parts of most media can be acquired from the literature [31], and the refractive index of jet fuel is *n* = 1.45 near 940 nm. The imaginary part of the refractive index can be calculated by the transmittance.

The relationship between the imaginary part of the sample refractive index η and extinction coefficient *k* can be described as [32]:

(13) $$\eta =\frac{k\cdot \lambda }{4\pi }$$

where λ is the wavelength of the incident light in vacuum.

According to Lambert-Beer law, the relationships between the transmittance and the extinction coefficient is:

(14) $$lg\left(\frac{I_{0}}{I}\right)=D\cdot ln10=k\cdot h$$

where *I*_0_ is the initial incident light intensity, *I* is the transmitted light intensity, *D* is the optical density, and *h* is the distance of the light in the medium.

According to Equations (13) and (14), the imaginary part of the refractive index can be calculated as:

(15) $$\eta =\frac{lg\left(\frac{I_{0}}{I}\right)}{h}\cdot \frac{\lambda }{4\pi }$$

The Shimadzu UV-3101PC photometer was used to measure the transmittance of jet fuel. A square glass container with an inner wall length of 55 mm was used to store the jet fuel. After calibrating the photometer, the empty square glass container was placed in the effective detection area, and measured the transmittance of the empty glass container from 939.5 to 940.5 nm with interval of 0.05 nm. Then the glass container was filled with jet fuel and it was placed in the detection area and the transmittance curve of glass container filled with jet fuel was measured from 939.5 to 940.5 nm with an interval of 0.05 nm.

According to the measured transmittance of a glass container and a glass container filled with jet fuel, the imaginary part of the refractive index was 1.211 × 10^−7^ when the incident light is 940 nm according to Equation (15). So that the complex refractive index of the jet fuel in this paper is m = 1.45 − 1.211 × 10^−7^ i.

### 3.2. Simulationprocedure

**Figure 3:**
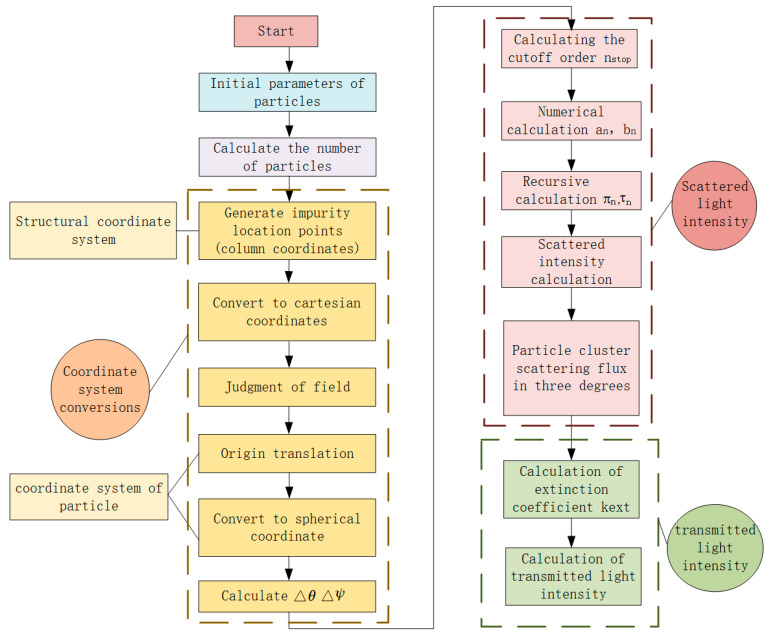
Simulation flow chart.

A program was written to calculate the scattered light energy and transmitted light energy of jet fuel at multi-angles during the experiment. The flowchart of the numerical calculation was shown in Figure 3. It was mainly divided into three parts, coordinate system conversion, calculation of scattered light intensity and calculation of transmitted light intensity. The input to the program including the particle information, including particle size distribution, mass concentration, refractive index and density, was determined and then the number of particles in the sample pool was obtained.

**Figure 4:**
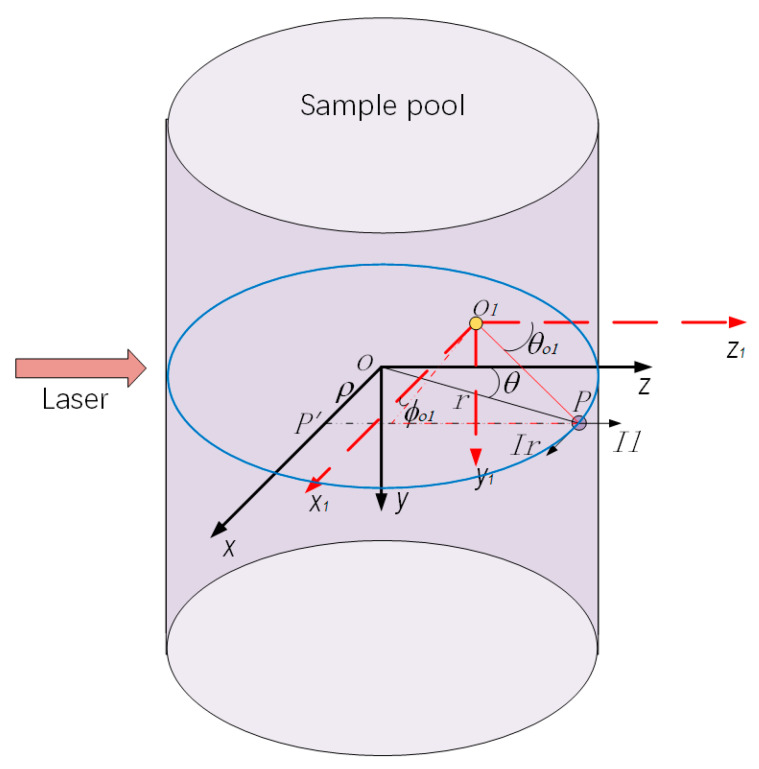
Diagram of the coordinate system.

First, we established and converted the coordinate system. The coordinate system was shown in Figure 4. The structure coordinate system was a Cartesian coordinate system with the intersection of the sample pool axis and the optical path axis used as the coordinate origin, the light direction as the positive *Z*-axis direction, the height direction from the rotating blade to the mouth of the container as the positive *Y*-axis direction, and the positive *X*-axis direction determined according to the right-hand rule. However, the Mie scattering was calculated in a spherical coordinate system with the scattering particle as the origin, and the coordinates of the receiving surface of the scattered light also need to be expressed using this coordinate system. Since both the origin and the type of coordinate system were different between the Mie scattering calculation coordinate system and the prototype structure coordinate system, a coordinate conversion was required.

In the structure’s cylindrical coordinate system, n particles were randomly generated in the optical path range and their coordinates were recorded. Then we converted the coordinates of the particles into structure coordinates in preparation for changing the origin of the coordinate system. Then the effective fields of the three scattering measurement detectors were judged respectively in order to select the particles within the detector field for the next step of calculation. Subsequently, the operation was transferred to the Mie scattering coordinate system with the scattering particle as the origin, and the range of scattering angle Δθ and azimuth angle Δφ were finally calculated.

In the calculation of scattered light intensity, firstly, the scattered light intensity of each particle at three angles was calculated, and then the scattered light intensity of each particle was summed to obtain the total scattered light intensity of the particle swarm. Several core scattering parameters in the calculation process were calculated as follows, according to Wiscombe’s empirical formula, the cutoff order *n_stop_* [33] was calculated, the Mie scattering coefficients an and bn were calculated using numerical calculations, and $\pi_{n}$ and $\tau_{n}$ were calculated using the recurrence method. Then, the scattering intensity functions *i*_1_ and *i*_2_ were obtained. As a result, the scattering intensity functions of the particle swarm were calculated, and the scattering light flux of the particles at the three angles was calculated.

Transmitted light intensity was calculated using the extinction method. After calculating the extinction coefficient of the particles, the transmitted light energy was then calculated using the Lambert-Beer law.

### 3.3. Scattering Characteristics Analysis

We used five solid impurity particles commonly found in jet fuels to investigate the relationship between the intensity of the scattered light and the scattering angles, or azimuth angles. For the five kinds of particles, we selected ${\mathrm{Al}}_{2}O_{3}$ particles, ${\mathrm{Fe}}_{3}O_{4}$ particles, Cu particles, $H_{2}O$ particles and polystyrene microsphere (PLS) particles. The complex refractive index and density of the five particles at 940 nm are given in Table 1. ${\mathrm{Al}}_{2}O_{3}$ particles, $H_{2}O$ particles and PLS particles belong to non-dissipative media, so the refractive index is a real number. However, ${\mathrm{Fe}}_{3}O_{4}$ particles and Cu particles are dissipative media that absorb light, so the refractive index is a complex number. It can be observed that the imaginary part of the Cu particle is larger than the real part.

**Figure 5:**
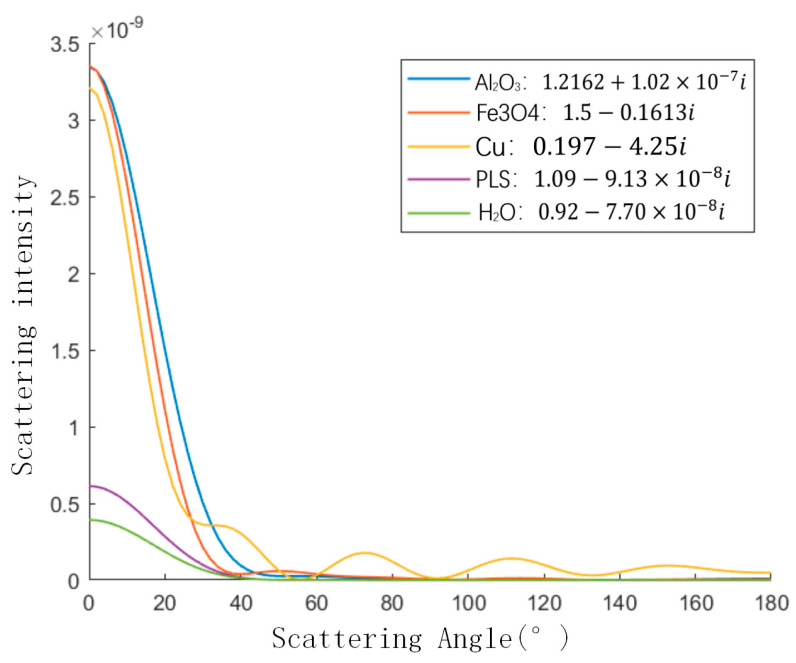
The relationship between scattering angle and scattering light intensity of different particles (α = 5, φ = 0).

Figure 5 shows the relationships between the relative intensity of scattering and the scattering angle for ${\mathrm{Al}}_{2}O_{3}$ particles, ${\mathrm{Fe}}_{3}O_{4}$ particles, Cu particles, $H_{2}O$ particles and PLS particles in a homogeneous jet fuel with the particle size parameter *α* = 5 and azimuthal angle *φ* = 0. Where $\alpha =m_{1}\pi d/\lambda$, $m_{1}$ is the complex refractive index of the medium, *d* is the particle size and λ is the wavelength. It can be seen that for different impurity particles in aviation kerosene, the distribution curves of scattered light intensity with scattering angle have similar trends when the particle size parameters are the same. The scattered light intensity tends to be stable when the scattering angle is about 40°. The starting values of ${\mathrm{Al}}_{2}O_{3}$ particles, ${\mathrm{Fe}}_{3}O_{4}$ particles, and Cu particles are of the same order of magnitude, as those of $H_{2}O$ particles and PLS particles. The scattered light intensity of Cu particles fluctuates because the absorption coefficient in the relative complex refractive index of Cu particles in jet fuel is larger.

**Figure 6:**
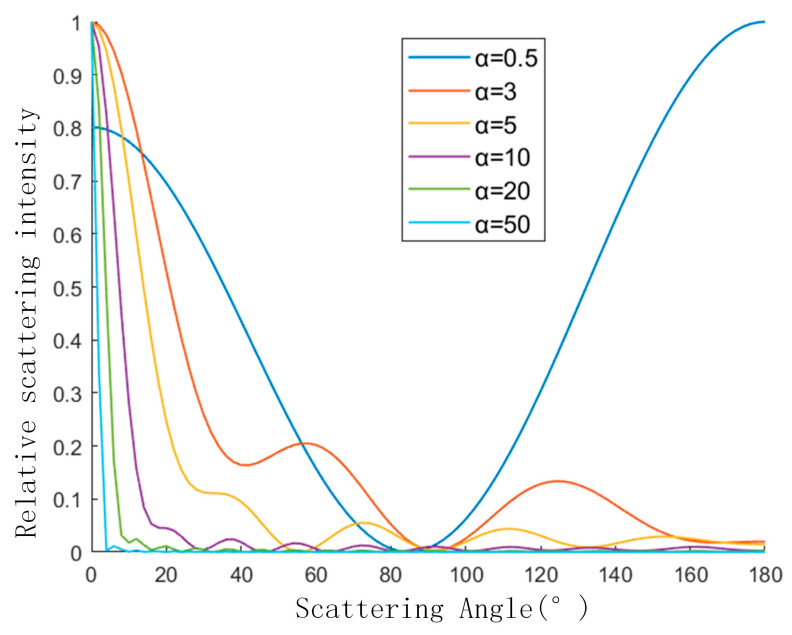
The relationship between scattering angle and scattering light intensity of different sizes copper particles.

Figure 6 shows the variation curve of scattered light intensity with the scattering angle of copper particles in aviation kerosene at different particle sizes. Since the order of magnitude of scattered light intensity varies greatly, the scattering intensity is normalized for convenience. When α = 0.5, the curve shows the law of Rayleigh scattering, and the forward scattering and backward scattering are similar. The more rapidly the scattered light falls in the forward small angle as particle size increases, the more the scattered light intensity is concentrated in the forward small angle.

**Figure 7:**
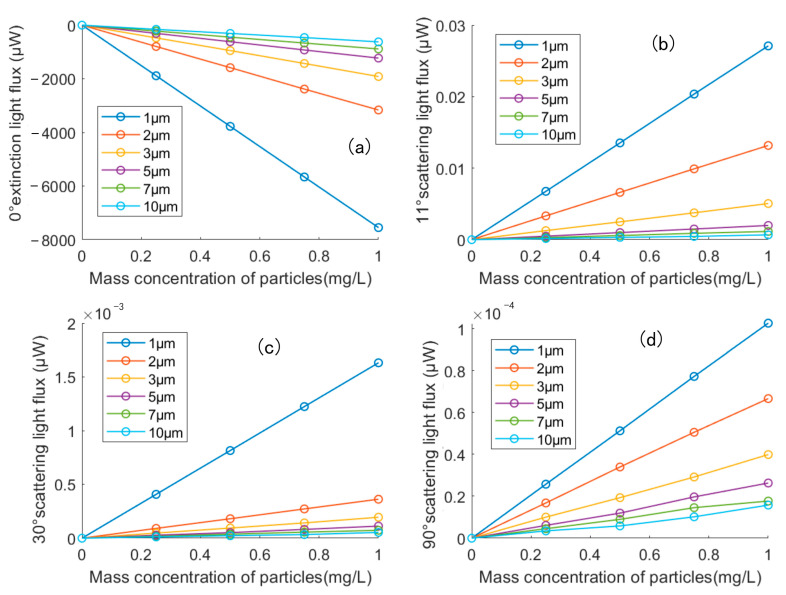
The relationship between the mass concentration of copper particles and the light flux at angle (**a**) 0° (**b**) 11° (**c**) 30° (**d**) 90°.

The results of the numerical simulation of the copper particle swarm are shown in Figure 7. Figure 7a showed the transmitted light flux, which was calculated by Lambert-Beer Law at different copper particle mass concentrations when the particle sizes of copper particles were 1 µm, 2 µm, 3 µm, 5 µm, 7 µm and 10 µm. It can be seen that the relationships between particle mass concentration and transmitted light flux attenuation is linear. When the particle mass concentration gets larger, the transmitted light flux attenuation of 0° will increase. Figure 7b–d were the scattered light fluxes using Mie scattering theory at scattering angles of 11°, 30° and 90°. It can be found that the higher the mass concentration of particles, the larger the scattered light flux will be. The scattered light flux at the same mass concentration became smaller with increasing particle size.

**Figure 8:**
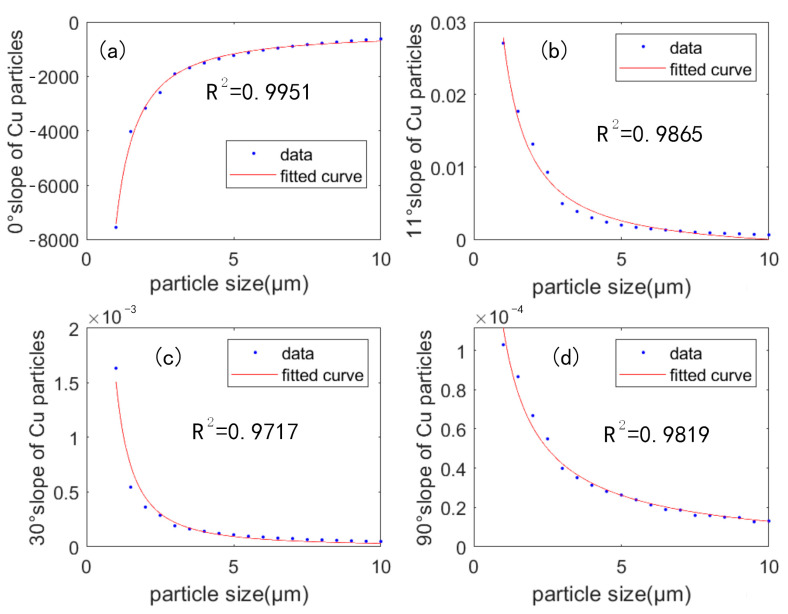
The relationship between the slope and the copper particles size at angle (**a**) 0° (**b**) 11° (**c**) 30° (**d**) 90°.

Figure 7 shows that the mass concentration of copper particles was linearly related to the transmitted light flux attenuation and the scattered light flux. The most important parameter in the linear relationship was the slope, and the slope curves of the light flux-mass concentration relationship at different copper particle sizes were shown in Figure 8. Figure 8a showed the fitted relationship for the 0° absorption channel. It can be seen that the trend of the curve is exponentially increasing. The larger the particle size, the smaller the transmitted light flux attenuation will be. The transmitted light flux was closer to the initial light intensity. Figure 8b–d show the fitting relationships between the particle size and the scattering light flux slope at three angles, respectively. It can be seen that the trend of the curve is decreasing exponentially. The variation in scattered light flux decreases as the particle size increased. The coefficient of determination (referred to as R^2^) was greater than 0.97, which means the fit is great.

## 4. Experiment

This paper has developed a prototype to measure laser transmitted light intensity and scattered light intensity at three angles in order to investigate the relationships between mass concentration and particle size on multi-angle scattered light intensity and transmitted light intensity in large samples with dynamic measurements.

### 4.1. Prototype Setup

**Figure 9:**
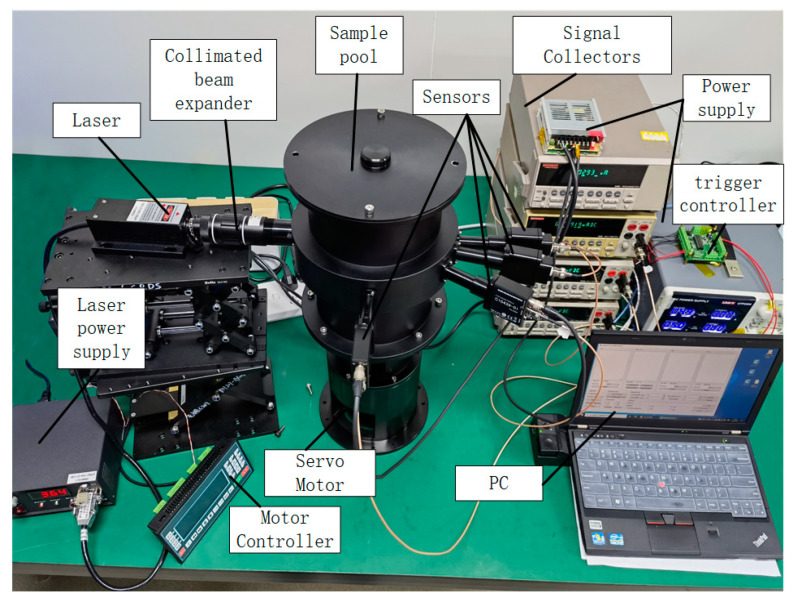
The drawing of the experimental installation.

**Figure 10:**
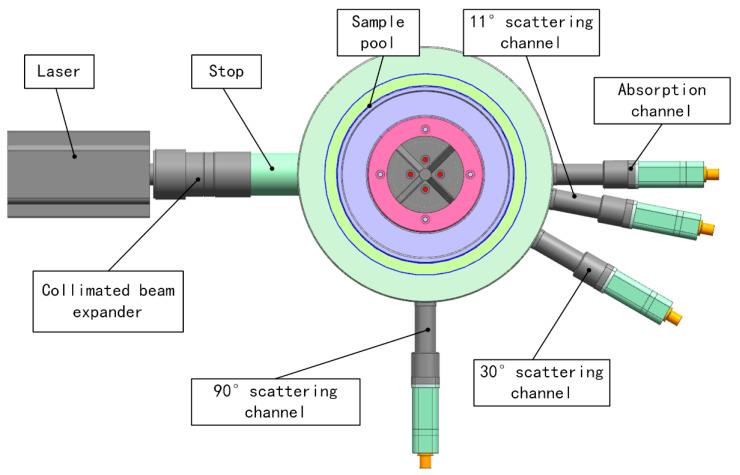
The drawing of the optical system.

The prototype was shown in Figure 9. The equipment is mainly composed of a laser, a sample pool, sensors, a motor, a motor controller and driver, several power source, multimeters, an external trigger controller and a computer. The sample pool was a cylindrical cavity with a diameter of 150 mm and a height of about 230 mm. The laser path length was about 150 mm. Scattering and absorption of particles occurred inside the sample pool. There were one laser incidence window and four detector receiving windows on the wall of the sample pool. Figure 10 shows the angular relationships between the detectors. The incident window and the 0° detector window were in the same line, and the angles between the optical axis of the other three detector windows and the axes of the 0° window were 11°, 30° and 90°. The window glasses were made of fused quartz, and coated on both sides with an anti-reflective film that had a transmittance greater than 99% at 930–950 nm.

Since settling of the solid particles mixed in the jet fuel may occur when the fuel was left to stand, bladed for stirring the sample were added to the bottom of the sample pool to prevent settling of the particles. Different rotational speeds were also used to simulate scenarios with varying flow rates. Servo motors were excellent at controlling speed and position accuracy, so a servo motor (YIXING, Servo Motor 80) with a rated torque of 2.4 N·m and a rated power of 750 W was used as the power source of the stirring vane. The motor was controlled by an integrated controller based on a PLC and driven by a motor driver.

### 4.2. Optical System

The optical system is shown in Figure 10. The laser (CNI, MDL-III-940L) was used as the light source for particle detection. Its emission wavelength was 939.87 nm with a spectral bandwidth of 0.8 nm, a divergence angle less than 1 mrad, and a power stability within 4 h better than 1%. The light emitted by the laser was a collimated beam with a diameter of 10 mm after the beam expander. Collimated light entered the sample pool through the fused quartz window after passing the anti-stray stops. The light was scattered and absorbed by the particles in the sample pool. The transmitted light passed through the exit window and was focused on the detector by the lens. The intensity of the scattered light was scattered at various angles. We had three scattering detector assemblies at 11°, 30° and 90° that can receive the light scattered by the particles. Photodiode modules (HAMAMATSU, C10439-01, Japan) with an effective surface size of 2.4 × 2.4 mm were used in the 30° and 90° directions and had high sensitivity in the visible to near-infrared range, low dark current, and high reliability. Silicon photodiodes (HAMAMATSU, S2386-5K) were used in the 0° and 11° directions.

### 4.3. Data Acquisition System

The prototype needed to acquire light flux at different angles at the same time. The solid impurity particles during the experiment were non-static, so the data acquisition system was required to collect 4-channel data simultaneously to ensure that the 4-channel data were the light intensity which the measured sample was in the same state. This prototype used external triggering to ensure time synchronization.

The synchronization of the detector data acquisition system was achieved using the external trigger mode of a multimeter (Keithley, 2000 or 6485, Solon, OH, USA). A self-developed external trigger controller was used to send control signals to the four multimeters to ensure that they can perform data acquisition simultaneously. The main controller of the data acquisition system was a computer, which used five RS-232 serial ports to control the external trigger and collect data from the whole prototype. Serial ports 1–4 were connected to four multimeters, and the data cached after the trigger can be read through serial communication. Serial port 5 was connected to the external trigger controller for controlling it.

**Figure 11:**
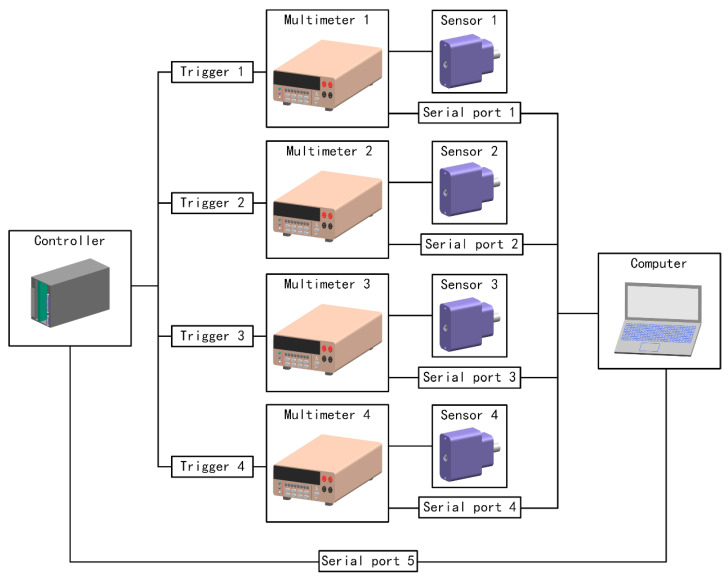
The drawing of experimental data acquisition system.

The composition block diagram of the experimental data acquisition system was shown in Figure 11. The single measurement process started with the computer sending an external trigger control signal through serial port 5. Then after the external trigger control module receives the control signal, it sent a trigger signal to the four multimeters, and the multimeters then cache the current measurement results. Finally, the computer read the cache of each multimeter through serial ports 1–4, and the measurement finished after reading.

In fast measurement mode, the above measurement process would be repeated at an equal time interval. The trigger command would be sent after polling the data from four channels. Due to the limitations of communication baud rate and instrument response, the sampling interval was 50 ms during the actual test.

### 4.4. Experimental Sample

When adding solid particles to jet fuel (RP-3), we used a concentrated solution of solid particles in proportion to clean jet fuel. An ultrasonic vibrator was used to mix solid particles and jet fuel thoroughly. Then we used a pipette to add a certain amount of the mixed solution to the sample pool. Copper particles with average particle sizes of 500 nm, 1 µm, 3 µm, 5 µm and 10 µm were selected as test samples. The mass concentration of copper particles in jet fuel is under 1 mg/L.

## 5. Discussion

**Figure 12:**
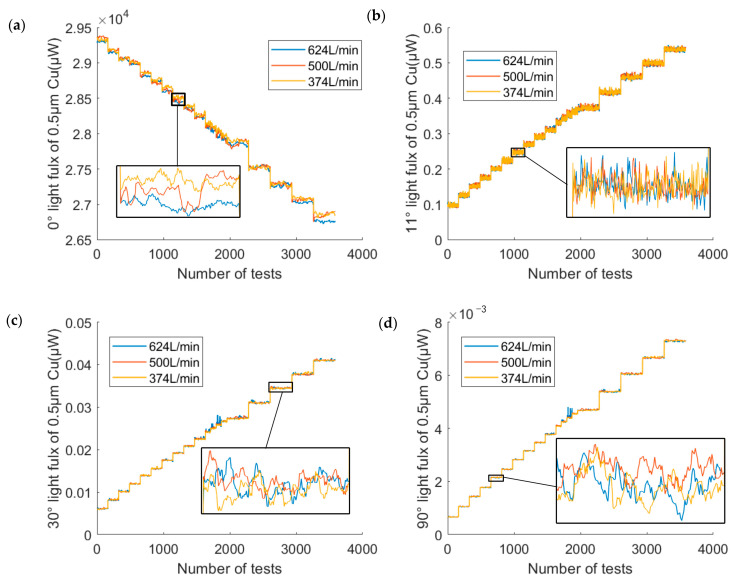
The data of 0.5 µm copper particles at different flow rates at (**a**) 0° (**b**) 11° (**c**) 30°and (**d**) 90°.

We measured the light signal of copper particles with different particle sizes and different mass concentrations using the above prototype. Then the scattered light flux and the transmitted light flux were calculated. During the test, the scattered and transmitted light fluxes at the motor speeds of 75 r/min,60 r/min and 45 r/min were measured. According to Equation (16), the equivalent flow rate is converted from rotational speed to 310 L/min, 250 L/min and 187 L/min, respectively. Figure 12 shows a comparison graph of the scattering measurement results for copper particles with an average diameter of 0.5 µm at three equivalent flow rates. It can be seen that the signal values of the four angles have little difference under the three flow rates, so the influence of the flow rate on the light flux is negligible when the flow rate was between 187 and 310 L/min. The higher motor speed had more benefit in avoiding settlement, so the following data was based on an equivalent flow of 624 L/min. Use equivalent flow rates to convert rotational flow rates to pipe flow rates. The equivalent flow rate is the same flow rate per unit time through the optical path for both dynamic methods. For the rotational dynamic mode, the flow through the optical path per unit time is:

(16) $$Q=\int_{0}^{\frac{D_{s}}{2}}D_{l}2\pi nD_{s}dD_{s}$$

where $D_{s}$ is the diameter of the sample pool, $D_{l}$ is the laser beam diameter, n is the speed of rotation of the liquid.

In the pipe flow dynamic mode of the pipe, when the flow rate through the optical path range is *Q*, the equivalent flow rate $E_{v}$ of the fluid in the pipe is:

(17) $$E_{v}=\frac{\pi D_{s}Q}{4D_{l}}$$

**Figure 13:**
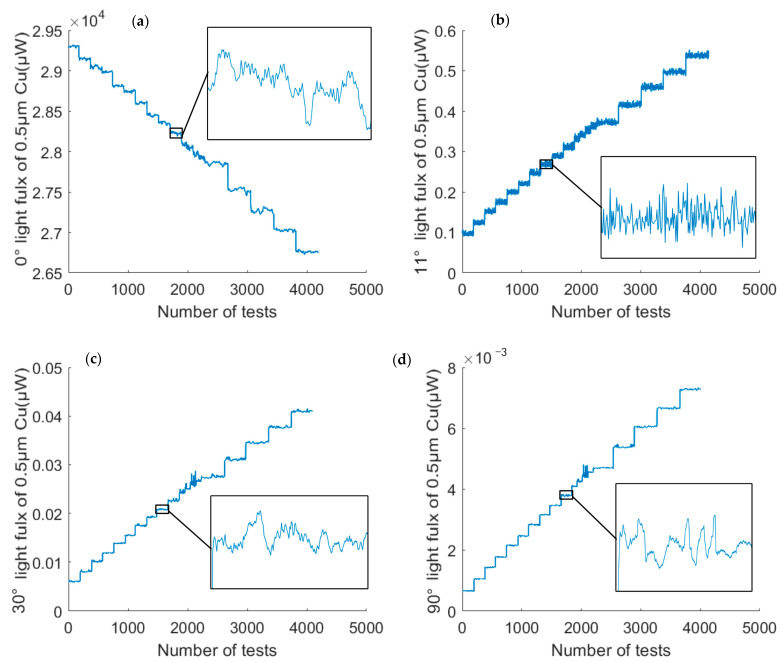
Measured data of 0.5 µm copper particles at (**a**) 0° (**b**) 11° (**c**) 30° and (**d**) 90°.

Figure 13 shows the experimental data for four angles with a particle size of 0.5 µm. In the experiment, 200 sets of data were acquired at 50 ms intervals for each mass concentration. It should be noted that the “height” of the light flux steps in the figure was different because the intervals of the mass concentrations were not chosen uniformly during the experiments.

**Figure 14:**
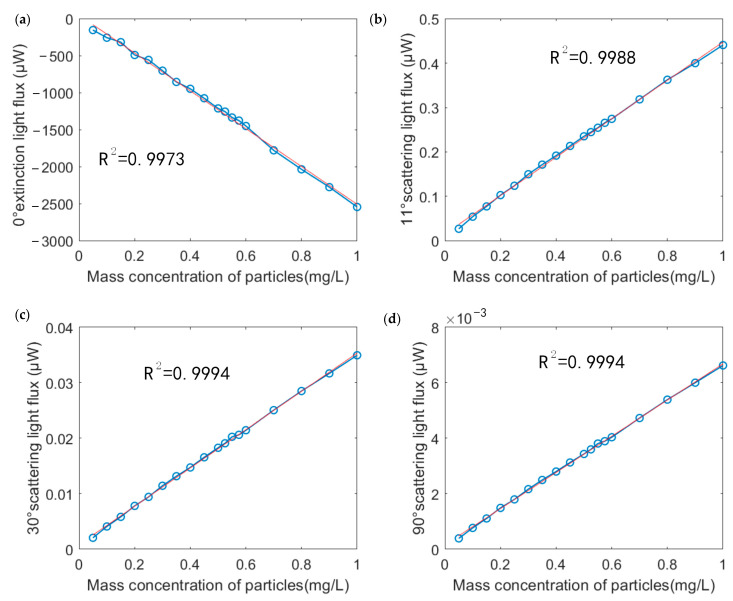
The fitting of the mass concentration and light flux of 0.5 µm copper particles at (**a**) 0° (**b**) 11° (**c**) 30° and (**d**) 90°.

After Pauta criterion screening for 200 data points in each group, the average value of each group’s data was calculated. The relationship between the light flux of every channel and the particle mass concentration was drawn in Figure 14, and the linear fitting was carried out. As seen in Figure 14, the collected data at different mass concentrations had a good linearity, and the linearity of the fitting R^2^ was better than 0.99.

**Figure 15:**
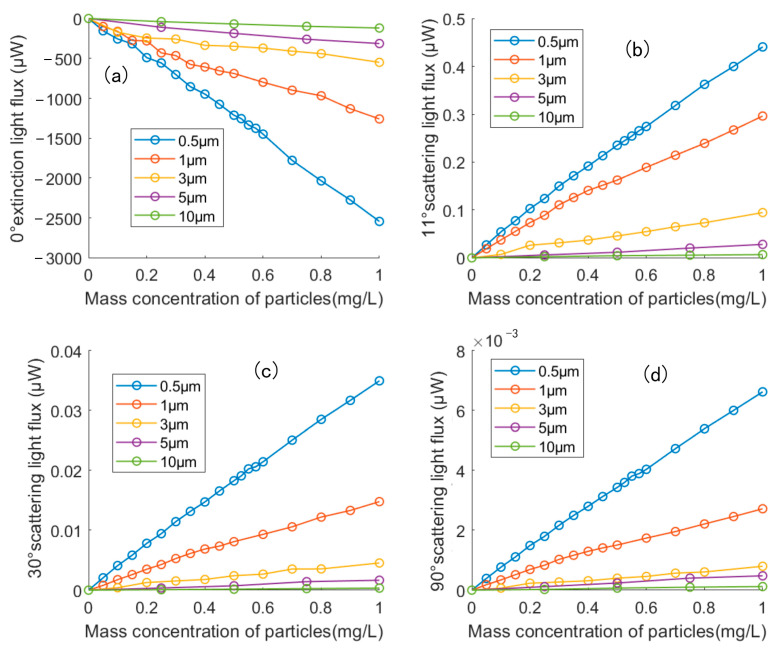
The relationship drawing of mass concentration and light flux of copper particles with different particle sizes at (**a**) 0° (**b**) 11° (**c**) 30° and (**d**) 90°.

In addition to 0.5 µm copper particles, experiments were also performed based on the same method using copper particles with average particle sizes of 1 µm, 3 µm, 5 µm, and 10 µm. Figure 15a shows the relationship between particle mass concentration and transmitted light flux attenuation for copper particles of different average particle sizes. For different diameters of copper particles, the experimental data showed good linearity: the transmitted light flux decreases with the increase in particle size with the same particle mass concentration. Figure 15b–d show that the scattered light flux decreases as particle size increases while particle mass concentration remains constant. The above experimental results were similar to the theoretical calculations and had the same trend of variation.

**Figure 16:**
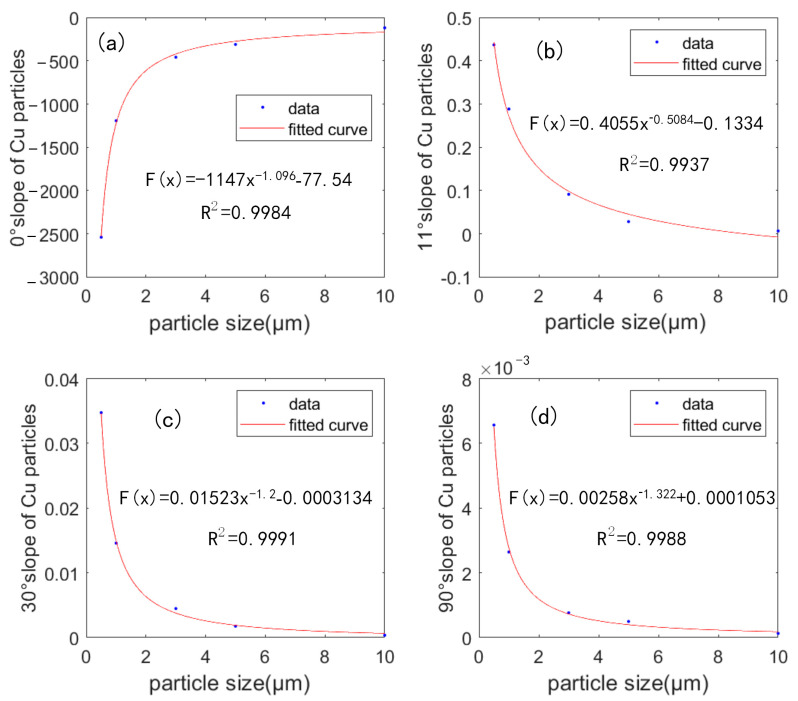
The fitting drawing between slope and particle size of copper particles with different sizes measured at (**a**) 0° (**b**) 11° (**c**) 30° and (**d**) 90°.

The slope of copper particles with average particle size of 0.5 µm, 1 µm, 3 µm, 5 µm and 10 µm was fitted exponentially to the equivalent particle sizes. The most important parameter in the linear relationship was the slope, and the slope curves of the light flux-mass concentration relationship at different copper particle sizes were shown in Figure 16. The R^2^ was above 0.99 and the trend of variation was similar to that of the simulation results, which was consistent with the theoretical calculation.

## 6. Conclusions

In this paper, the refractive index of the jet fuel used in the experiments had been determined by calculating the transmittance. The scattering characteristics of the copper particle swarm in jet fuel had been analyzed by numerical simulations in this test equipment. It was found that the relationships between the mass concentration of the particle swarm and transmitted light flux parameter and luminous flux should be linear. There were exponential relationships between signal increment and particle size at unit mass concentration. Finally, the self-built prototype had been used to validate the calculation method of the particle swarm’s transmitted and scattered light intensity based on Mie scattering and the Lambert-Beer law. The prototype of dynamic measurement in a large sample designed in this paper can characterize the average particle size and mass concentration of particles in jet fuel by collecting and calculating the signal values of four channels and processing them. It has provided a theoretical and experimental basis for full-flow online measurement of impurity particles in jet fuel. We intend to use this principal prototype to conduct the next research direction to invert the relative refractive index by the relationship between the signal intensity between the four channels. The equipment for full-flow online measurement of impurity particles in jet fuel will be further studied in the future.

## Figures and Tables

**Table 1:** Complex refractive index and density of five solid impurities.

Particles	Complex Refractive Index (940 nm)	Density (mg/mm^3^)	Reference
${\mathrm{Al}}_{2}O_{3}$ particles	1.7569	3.99	Malitson et al. 1972 [34]
${\mathrm{Fe}}_{3}O_{4}$ particles	2.168–0.223i	5.18	Querry 1985 [35]
Cu particles	0.28419–6.1375i	8.96	Rakić et al. 1998 [36]
PLS particles	1.5739	1.06	Sultanova et al. 2009 [37]
$H_{2}O$ particles	1.3274	1	Hale et al. 1973 [38]

## Data Availability

No new data were created or analyzed in this study. Data sharing is not applicable to this article.
